# Toward the validation of VR-HMDs for medical education: a systematic literature review

**DOI:** 10.1007/s10055-023-00802-2

**Published:** 2023-05-13

**Authors:** Shiva Pedram, Grace Kennedy, Sal Sanzone

**Affiliations:** 1 SMART Infrastructure Facility, Faculty of Engineering and Information Sciences, University of Wollongong, Wollongong, Australia; 2 Faculty of Science, Medicine and Health, School of Medicine, University of Wollongong, Wollongong, Australia

**Keywords:** HMD-VR, Virtual reality, Immersive technology, Surgical training, Medical training, Systematic review, Requirement, Validation

## Abstract

The latest technological advancements in the domain of virtual reality (VR) have created new opportunities to use VR as a training platform for medical students and practitioners more broadly. Despite the growing interest in the use of VR as a training tool, a commonly identified gap in VR-training for medical education is the confidence in the long-term validity of the applications. A systematic literature review was undertaken to explore the extent of VR (in particular head-mounted displays) applications for medical training with an additional focus on validation measures. The papers included in this review discussed empirical case studies of specific applications; however, these were mostly concerned with human–computer interaction and were polarized between demonstrating that a conceptual technology solution was feasible for simulation or looked at specific areas of VR usability with little discussion on validation measures for long-term training effectiveness and outcomes. The review uncovered a wide range of ad hoc applications and studies in terms of technology vendors, environments, tasks, envisaged users and effectiveness of learning outcomes. This presents decision-making challenges for those seeking to adopt, implement and embed such systems in teaching practice. The authors of this paper then take a wider socio-technical systems perspective to understand how the holistic training system can be engineered and validated effectively as fit for purpose, through distillation of a generic set of requirements from the literature review to aid design specification and implementation, and to drive more informed and traceable validation of these types of systems. In this review, we have identified 92 requirement statements in 11 key areas against which a VR-HMD training system could be validated; these were grouped into design considerations, learning mechanisms and implementation considerations.

## Introduction

Over the last decades, computer-assisted training has gained significant momentum (Jou and Wang 2012). More specifically, technology-mediated learning has become increasingly popular among high-risk industries such as defense, aviation and medicine (Mehrotra and Markus 2021a). In the field of health, technology-mediated learning has been defined as the learning platform in which participants’ interaction with other participants, objects within the environment or educator is mediated through technology (Alavi and Leidner 2001) which allows learners to repeatedly practice without the risk of error causing detrimental effects in an actual patient while enhancing their clinical skills and efficiency. Virtual reality (VR) is a defined as a computer-generated reality, which creates an opportunity for learners to experience various auditory and visual stimuli experienced through specialized hardware, such as head-mounted displays (HMDs) (INACSL Standards Committee 2016). The implementation of VR in medicine is not new and has been used in various medical contexts, ranging from nursing, clinical psychology, hospital teamwork, anatomical discovery, surgical operational training, pre- and post-surgical training, and for augmentation, and has shown increasing potential for improving learning outcomes (Vaughan et al. 2016; Bracq et al. 2021; Mehrotra and Markus 2021a).

The advances in VR technologies, increased availability and reducing hardware costs have diminished many of the early challenges over adoption of VR. Alongside technological development, the landscape of educational systems has endured a period of upheaval and disruption of teaching due to the COVID-19 pandemic which in many countries worldwide caused varying levels of lockdown and necessitated shifts to remote forms of learning. Together, these two factors have led many to view VR as an enticingly viable proposition for training. There are, however, still many challenges around how to measure and attain learning outcomes so that VR solutions can be demonstrated to actually be able to deliver true value and enhancement over traditional means. Recent reviews in the field of VR training show that there has been an increase in studies; however, the focus of learning outcomes and contexts have been ad hoc, and as such, the genericity of published solutions is insufficient for wide-scale adoption across the gamut of training purposes (Renganayagalu et al. 2021).

This paper seeks to increase understanding around the current applications of VR-HMD training (for medical contexts) and outlines an aggregated set of generic requirements for VR training within medical education systems. A systematic literature review was performed with two related research questions in mind:

1. What do we know about the use of VR-HMDs for teaching clinical skills to medical students or practitioners?
2. What are the requirements for such a VR training system?

The structure of the paper is as follows: firstly, the scope of the VR-HMD training system is introduced from the micro-level (involving individual person to technology interactions) and widened to the meso-level encompassing the wider educational training ecosystem this sets the scene for the system of interest. The methodology used for the systematic literature review is briefly described before the summary analysis and findings from the review are laid out. A set of requirements for VR training is then collated from the review, and finally, a hierarchical requirements framework is proposed.

## Scope of the VR-HMD training system for medical education

Micro-, meso- and macro-level frameworks have been used extensively within healthcare to understand problems and complex systems from varying strata perspectives (World Health Organization 2002). Within the context of VR-HMD training, the micro-level concerns the (simulated) patient interaction level. Investigating micro-level interactions alone will not yield validation of long-term training because medical education training systems consist of several strategies (not just VR exclusively); therefore, overall training outcomes should be considered at the meso-level (the education provider organization).

**Fig. 1:**
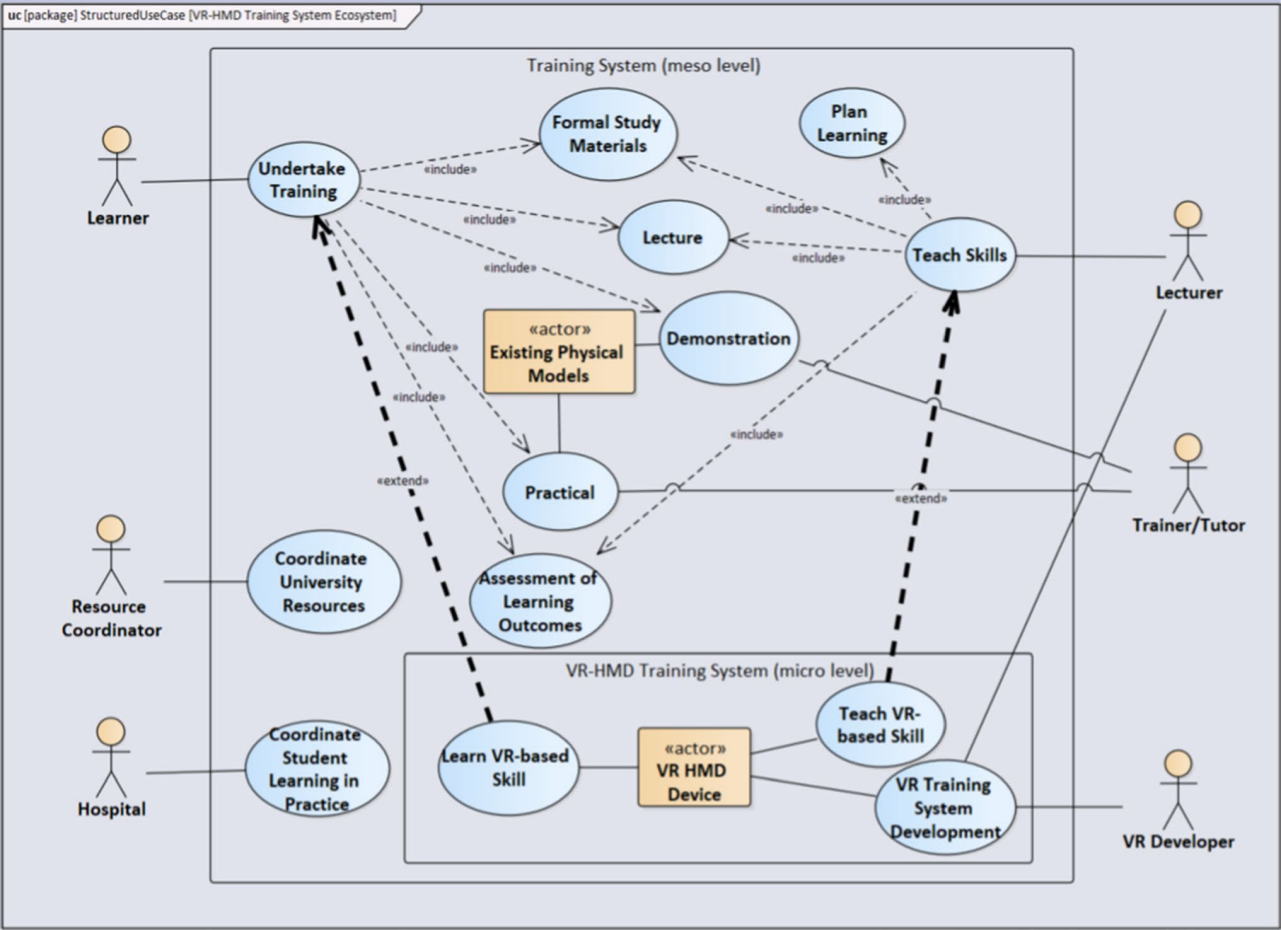
VR-HMD training system ecosystem (use cases)

While the recent body of literature around VR-HMD training systems is heavily descriptive of the system being synonymous with the technology and composed entirely of hardware/software elements, the authors of this paper take a wider socio-technical systems perspective to understand how the holistic training system (constrained to VR-HMDs) can be engineered and validated effectively as fit for purpose. Engineered systems can be consisted of any of all of people, products, services, information, processes and natural elements (Sillitto et al. 2019); in the case of our training system of interest this includes the technology, but also the people (from the learners, to the trainers, and the VR developers), the information being communicated and the wider processes or tasks being undertaken within the VR simulation. This paper also considers the wider ecosystem in which the VR training system (micro-level) is intended to be deployed, which is the wider training system (meso-level) which in turn has its own set of processes and resourcing in which the VR training must integrate. Figure 1 shows a generic set of stakeholders (actors) and their envisaged use cases involved in the VR-HMD Training System Ecosystem at the point of VR-HMD introduction. In most cases, the meso-level training system is existent before the introduction of the VR-HMD training system, so the use cases are likely to be extensions of the existing training use cases (shown as bold dashed <  < extend >  > lines). In the existent training system, the generic use cases show the primary use cases of the learner undertaking training, and the lecturer (alongside tutors/trainers) providing the teaching of skills. The <  < include >  > relationships depict sub-use cases; for example, ‘Undertake Training’ includes the study of formal materials (such as textbooks, handouts, videos), attending lectures, demonstrations, practical, and completing assessments against the learning outcomes. The ‘Teach Skills’ side of the relationship maps across to the same use cases but from the provision and delivery side.

## Method

**Fig. 2:**
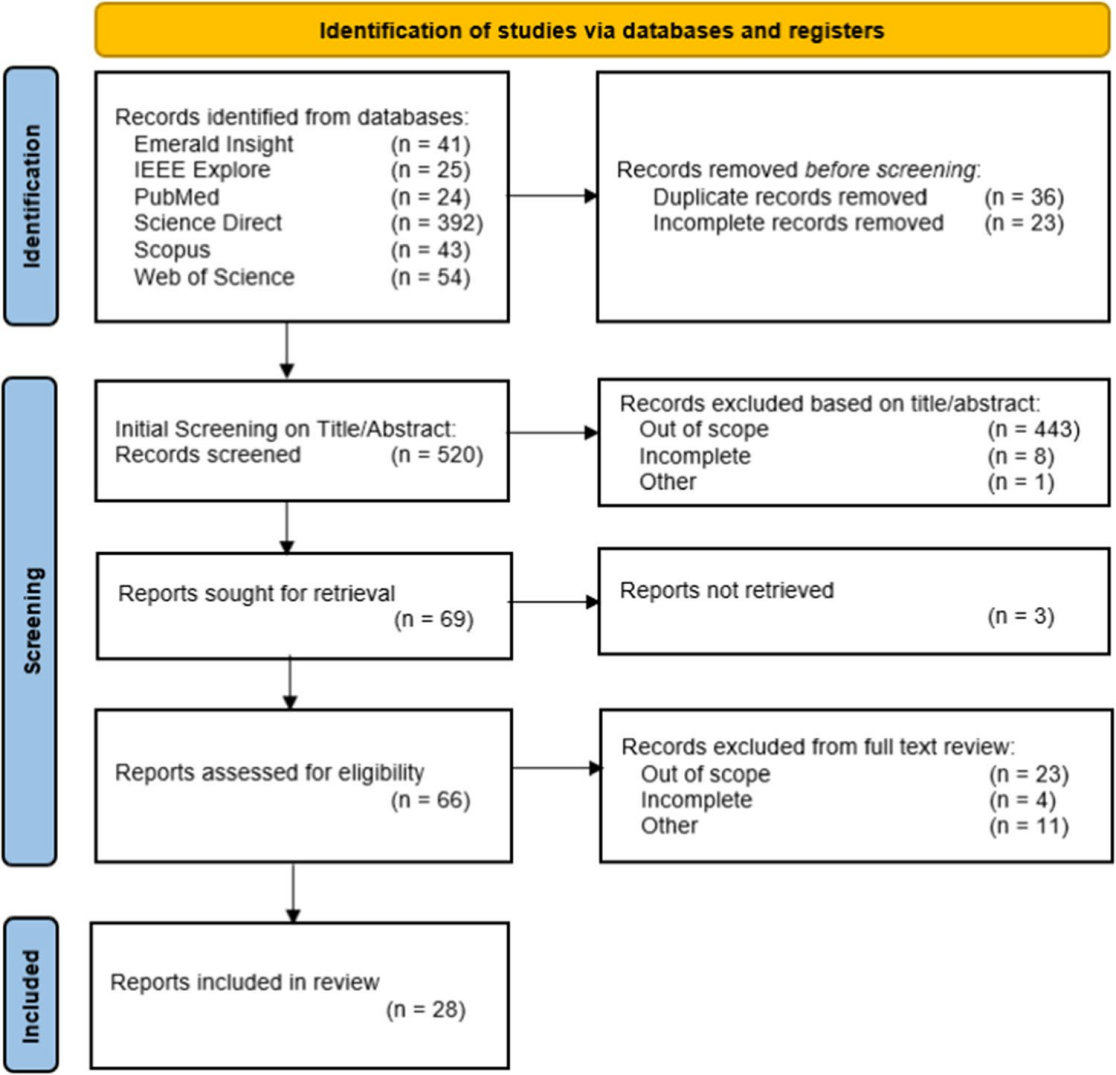
PRISMA flow diagram record

A systematic search of the literature was performed to identify how VR-HMDs have been used for teaching clinical skills to medical students or practitioners and what are the requirements for successful training delivery. The Preferred Reporting Items for Systematic Reviews and Meta-Analyses (PRISMA) reporting method (Page et al. 2021) was used in order to ensure consistent capturing of information. Figure 2 shows the complete PRISMA Flow Diagram (according to the PRISMA 2020 Statement format) for this review.

A systematic literature search was performed in six electronic databases: PubMed, Emerald Insight, IEEE Explore, Science Direct, Scopus and Web of Science, in September 2021. The following key words were selected: HMD AND "Virtual Reality" AND (Surgery OR Medical) AND ("Education" OR "Training"), and the time limit applied was from 2016 to 2021. As a result, a total of 579 results were found and, after removing duplicates and incomplete paper, we end up with 520 papers. The authors then screened the abstracts of all the remaining papers for full-text review eligibility. The eligibility criteria were:

- The papers had to be written in English
- Only original research articles, reviews and peer-reviewed proceedings were considered
- The training simulation must be relevant to all fields of surgical training such as operating room preparation, catheter insertion, injection, pre-operation planning or other relevant topics.
- The context of the scenario must be educational context, and the simulation scenarios had to be clearly explained.
- Furthermore, VR simulations were required to use a head-mounted display.

After Abstract review, we selected 69 papers for full text revision, and we identified 28 papers to be relevant to our research questions.

## Dataset analysis

### Network mapping

**Fig. 3:**
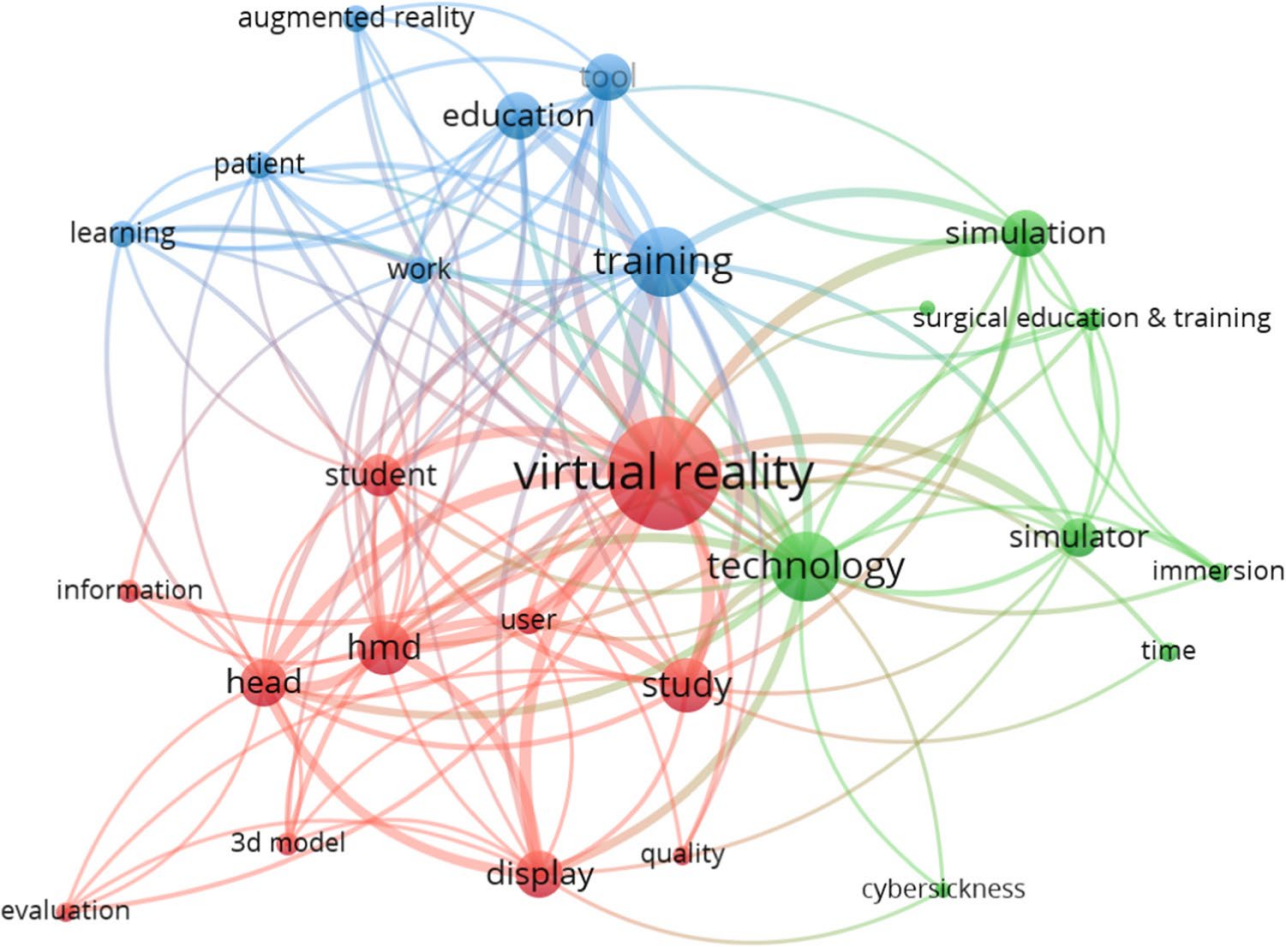
Bibliographic co-occurrences network map

Using the set of reports selected for qualitative review, a network map was created using VOS Viewer (v1.6.15), a bibliometric text mining visualization tool developed by van Eck and Waltman (2010). A keyword co-occurrences map was developed based on the frequency of keywords found in both the *title* and *abstract* data for all the included reports. After combining similar keywords via the thesaurus import function, the tool found 28 individual keywords with at least 4 occurrences across the data set. Figure 3 shows the network visualization of these keywords, with the size of the node representing the number of occurrences (in this case “virtual reality” has the largest circle and is, as to be expected, the most common occurrence being in all the records from the data set). In addition, the tool also derives co-occurrences (i.e., where keywords are found co-occurring in the same report), for this study, a threshold of 4 co-occurrences was used between terms (meaning that two particular keywords have been found co-occurring in at least 4 records). These co-occurrences are represented as the links between keyword nodes, the thicker the line, the more co-occurrences between those terms in the data set, the tool also represents tighter coupling between keywords as physically closer, and thus, keywords around the periphery tend to be outliers that are less co-occurring. Performing this bibliometric analysis gives an initial, objective idea of the nature of the keywords and how coupled certain terms are. The search terms naturally occur more frequently. Interestingly, the tool suggested three clusters: the red cluster tends toward the more physical aspects (e.g., *HMD, display, head, 3d model*), the green cluster seems to cover the technology and experience (e.g., *simulators, immersion, cybersickness*), and the blue cluster appears to be education and training. These clusters are not mutually exclusive; for example, one might reasonably expect *surgical education and training* to be in the blue cluster rather than green.

**Fig. 4:**
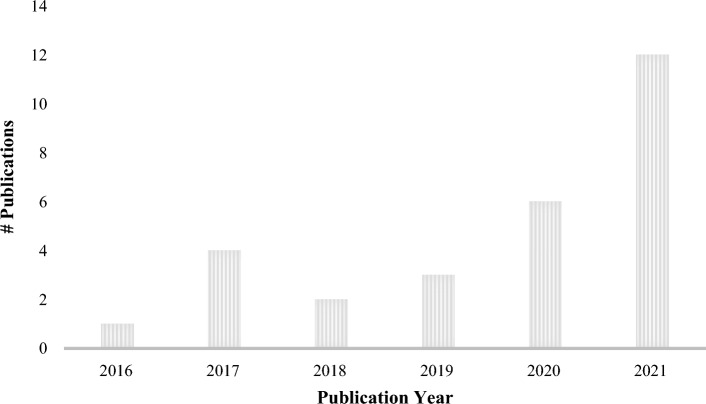
Publication date distribution

The publication date distribution shows a general trend (Fig. 4) with the exception of 2018; each year has shown an increase in publications on this topic. There is a skewing of the records with the majority of records (~ 42%) published within the 9 months prior to the literature search being performed. It is difficult to pinpoint the reasoning for this recent increase; however, the authors postulate that this large increase could be due to the greater availability of commercial VR solutions (such as Oculus, HTC Vive and Microsoft Hololens all released in 2016, with new models being released almost yearly since) along with the development of bespoke medical education VR solutions and subsequent lag for research progress with publication, or the resurgence of interest in VR due to remote learning imposed during the pandemic.

Out of 28 research papers, 14 papers were review papers elaborating on the application of VR in the surgical context and 14 papers were research designs focusing on providing the proof of concept for either pre-surgical or surgical training application (Appendix). Out of 14 research papers, 7 papers were single group studies meaning that all the subjects received a single intervention, and the outcomes were assessed based on their reports. The remaining six papers employed either within- or between-subject designs, where the impact of the interventions was assessed either between two groups or over time. Six out of 28 papers (21%) focused on reporting the application of VR as a pre-surgical training tool while 22 papers (78%) discussed VR as a procedural training platform (Table 1).

**Table 1:** Publication application and research type distribution

Research method	Pre-surgical training	Surgical training	Total
Between-subject design	2	4	6
Review paper	2	12	14
Single group	2	5	7
Within-subject design	0	1	1
Total	6	22	28

As it is summarized in Table 2, VR has been used in various context either as a pre-surgical training tool or as surgical training tool or both. This is a promising finding for the medical industry as it highlights the application of VR is not limited and can be used in various contexts.

**Table 2:** Context of use

Context of use	Pre-surgical training	Surgical training	Total
Anatomy	1	3	4
Cardiovascular training	0	2	2
Cranio-facial surgical training	0	1	1
Gynecology	0	1	1
Healthcare training	0	1	1
Laparoscopic surgery	0	2	2
Liver surgery planning application	1	0	1
Medical student training	0	1	1
Needle insertion training	0	1	1
Neurosurgery planning application	1	0	1
Neurosurgical training	0	1	1
Nurse training	0	2	2
Orthopedic surgical training	0	3	3
Plastic surgery	0	1	1
Resection planning	1	0	1
Sterile urinary catheter insertion	0	1	1
Surgery planning	1	0	1
Surgical instrumentation training	0	1	1
Surgical planning	1	0	1
Surgical skills acquisition training	0	1	1
Total	6	22	28

## Requirements for validation

A commonly identified gap in VR-HMD training for medical education is the confidence in the long-term validity of the applications (Lohre et al. 2020b; Mao et al. 2021b), in particular the acceleration of the learning curve (Huber et al. 2017a), efficacy of learning outcomes over time (Vaughan et al. 2016) and actual skills translation into real environments (Bielsa 2021). This paper has so far summarized the body of knowledge around VR HMD-based training systems within medical education. Subsequently, a second research question was addressed: “What are the requirements for a VR-HMD training system for medical education? “ Given the wide range of ad hoc applications and studies in terms of technology vendors, environments, tasks, envisaged users and learning outcomes, it is challenging for those seeking to adopt, implement and embed such systems in teaching practice. In order to support these decision-makers, a set of requirements was distilled from the literature review with a view to aiding design specification and implementation and to drive more informed and traceable validation of these types of systems. These requirements are not intended to be a prescriptive checklist, but a framework of requirements that should be considered and tailored for each training system being developed.

The set of reports studied in this systematic literature review were first revisited to elucidate the requirements that were either fulfilled by the VR system within that paper, or explicitly described in some form of a need. For each paper, textual searches were made for the keywords “need,” “must,” “shall” and “should” as indicators of need. The needs resulting from these searches were recorded, clustered and then grouped initially using an existing framework for VR training (Pedram et al. 2020) which describes seven key variables for effective technology-mediated learning using immersive VR (gaming & technology experience; participants state of mind; VR features; learning experience; usability; trainer & their feedback; and learning). It was found, however, that these variables did not provide sufficient scope for all the needs that had been recorded from the search.

**Fig. 5:**
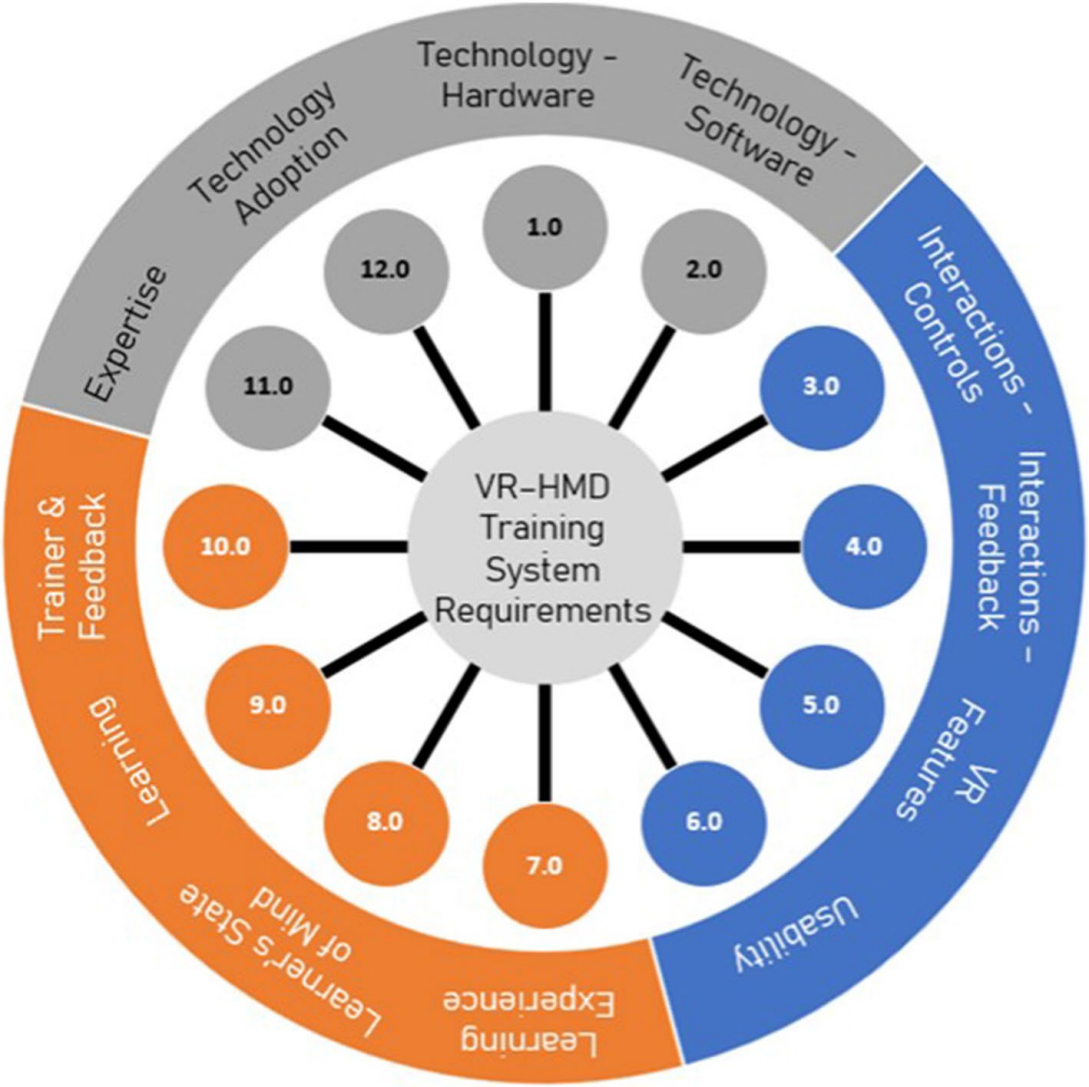
A Framework for VR training systems requirements

An extended framework is proposed in Fig. 5*.* The needs were translated into requirements statements, clustered across eleven broader categories (inclusive of the original seven) and logically grouped into three super-categories:

1. Design Considerations—the design of the VR simulation and synthetic environment.
2. Learning Mechanisms—the design of the learning that underlies the VR experience.
3. Implementation Considerations—the aspects that will impact the implementation of the design.

In total, ninety-two requirements statements were derived from the needs. These requirements are expressed discretely; however, there are relationships between them, both in terms of hierarchy and interactions. ISO 26800 (International Organization for Standardization 2011) describes the human–machine system model which, at its base micro-level, describes the simple interface between a human and machine performing a task within a widening scope of meso- and macro-environmental considerations. The key interactions between the human and machine involve the flow of information from the machine (displays) to the human (perception), and the actions performed by the human (effectors) on the machine (controls). Design of displays (or feedback) and controls for VR systems featured heavily in the requirements drawn from the literature.

### Design considerations

Design considerations for VR in medical education should prioritize educational accuracy, user proficiency, safety, usability and adaptability. The VR experience should accurately represent medical procedures, anatomy and physiology while being tailored to the skill level and learning objectives of the intended audience. Safety concerns, such as motion sickness or disorientation, must also be addressed. The design should be intuitive and user-friendly to facilitate ease of use and interaction with the content. Finally, the design should be scalable and adaptable to accommodate different learning objectives, medical specializations and technological advancements. In the next sections, the design considerations have been discussed.

#### Interaction: controls (1.0)

##### Gesture

*The system shall include gesture recognition*. Modern commercial VR-HMDs have hand-based controls that translate the user’s gestures and represent them within the synthetic environment. Better gesture control leads to more intuitive (Kenngott et al. 2021b) or natural (Lopes et al. 2017a) interactions.

##### Motion sensitivity

*The system shall provide motion sensitivity of controls*. Gesture and motion sensitivity are closely related in the creation of intuitive interfaces (Kenngott et al. 2021b). Interactions need to be precise and responsive to the users physical and corresponding virtual motions. In particular, motion control training is useful for manipulation of virtual objects and learning of psychomotor skills. Operations based on instinctual motion improve the learning experience (Lin and Yeh 2019).

##### Position tracking

*The system shall meet minimum position tracking thresholds*. For VR systems, the position of the user and dynamic changes in position are required to be tracked and translated into both the control and corresponding field of view such that the user feels immersed, but also to avoid mismatch between the real and perceived interactions. Pelargos et al. (2017b) identify substandard position tracking as a barrier to VR adoption in operative fields. There are a number of different technological solutions that could be used (most are integrated sensors in the HMD to provide inside-out positioning). For surgical applications, most skills require the use of handheld tools; therefore, the minimum position tracking capability should involve the head and two hand controllers.

##### Kinesthetic manipulation

*The system shall enable kinesthetic manipulation of virtual objects of interest*. Kinesthetic manipulation means that the user can control and move, inspect and manipulate virtual elements to better understand what the element is and how it works. Mäkinen et al. (2020b) discuss the use of manipulation of VR content via hand controllers as being beneficial to learning.

##### Movement efficiency and demands

*The system shall provide efficient movement controls*. The representation of movement of the controllers should be commensurate with the expected physical movements required of the task. Although Mao et al. (2021b) describe overall motion efficiency during tasks as a measure of training outcome, it is necessary to consider the design of the controls and layout such that it does not tax the user. Lopes et al. (2017a) describe the inadequacies of the design of the control tasks in a surgical instrument organization scenario, with users finding frequent movements between two areas separated by some distance causing discomfort.

##### Control element granularity

*The system shall provide control element granularity commensurate with the virtual layout needed*. Many surgical procedures involve fine movements and positioning; the VR control elements positional granularity must be at least equal to that required of the task (Lopes et al. 2017a).

#### Interaction: feedback (2.0)

##### Control feedback

*The system shall provide effective feedback to the user*. Having established that a set of controls is required, the corresponding part of the human–machine interaction loop is required to provide confirmatory feedback to the user that the appropriate controls have indeed been selected and to display the resulting changes and status of the system. It is important to consider feedback via an integrated combination of multi-model sensory channels (Vaughan et al. 2016).

##### Visual cues

*The system shall provide effective visual cues*. There were many studies that focused on the functionality of the HMD to provide visual cues that are appropriate to human vision and perception. Pelargos et al. (2017b) discuss the need for minimum thresholds for depth of field, depth of focus and field of view for the visual displays for immersion while others reported on experiments that considered the cognitive perception and understanding of how users respond to the visual cues. In synthetic environments, users tend to spend more time browsing the scene rather than specific controls (Lopes et al. 2017a; Matthews et al. 2021); this can result in inattentional blindness (difficulties in grabbing the attention of the user toward the virtual objects intended to be controlled) (McKnight et al. 2020a) and poses further challenges when gaze-based or eye-tracking controls are implemented (Lopes et al. 2017a).

##### Auditory cues

*The system shall provide effective auditory cues*. Sound-based feedback can be an artificial cue used to confirm that a task is completed (e.g., a confirmatory sound), or can be representations of natural sounds that the user would expect as realistic feedback specific to the scenario (Vaughan et al. 2016). Vaughan et al. (2016) suggest that multi-modal recordings of expert performance could be used to drive simulation design. This latter form of auditory realism is a particularly important contextual factor in medical education for scenarios where the users would use auditory cues as part of the diagnosis of a situation (Barteit et al. 2021b).

##### Haptic cues

*The system shall provide effective haptic cues*. The most commonly discussed limitations of the simulators for surgical procedure training are the lack of realistic tactile feedback (Mao et al. 2021b). VR to serve as fully comprehensive psychomotor skills training platform requires supporting haptic feedback. Generally, haptic sensory information can be tactile such as pressure on the skin, vibration, differences in temperature or force feedback such as sense the position and motion of pulse, and the forces exerted on the artery. It is then applied to the user as haptic feedback through the haptic device. Force feedback was most reported in studies, and there have been very few efforts to develop tactile feedback (Mäkinen et al. 2020). Haptic devices have several actuators to measure user's position and based on the context of use, block user's movement, giving an impression of force feedback. This is a gap in the existing settings and has proved essential in many scenarios.

#### VR features (3.0)

##### Co-presence

*The system shall establish a sense of co-presence in team-based training*. VR simulation is not constrained to single-user training but can be used to train team interactions and decision-making (Bielsa 2021). In addition, collaborative learning enables the sharing of knowledge through joint experiences. The pros and cons of team training should, however, be weighed up, Fairen et al. (2020) describe in their study of personalized VR teaching that students tended to prefer individual learning; however, the teachers in the study preferred a more collaborative learning approach.

##### Representational fidelity/realism

*The system shall provide representational fidelity/realism.* Three main types of fidelity were discussed in the papers: physical realism, procedural realism and contextual realism.

VR simulation has in the past primarily fixated on the fidelity of the physical representation of the patient/body models, from accurate anatomical and physiological models (Fairen et al. 2020; Kenngott et al. 2021b), kinematics (Vaughan et al. 2016), and dynamic patient interactions (Breitkreuz et al. 2021a). Conversely, Bernardo (2017) found that at the basic level, there was no significant difference in skills acquisition between simulated patient box-trainer models and VR simulation. The level of physical fidelity should be matched to the skill being acquired and is closely linked to the sense of immersion (Pedram et al. 2020).

In terms of skills acquisition, the VR system should also have appropriate levels of procedural realism so that skills learnt undertaking simulated tasks can be translated into real-life skills. Literature scans showed many instances of simulation of entire procedures (Huber et al. 2017a). Breitkreuz et al. (2021a) explored the use of a VR catherization game and discovered negative learning in one of the studied settings where the procedure is performed differently in practice. As well as providing a simulation of planned procedure, the simulations should also provide capability to display realistic cause and effects based on the actions of the user (Barteit et al. 2021b).

Contextual realism considers the recreations of aspects of the simulation other than the primary model and procedure of focus. The physical environment should match the given context. For example, if the procedure takes place in an operating theater, the simulated environment should be similar in terms of physical appearance and soundscape (Bernardo 2017).

##### Simulation purpose

*The simulation shall realize the benefits of VR over existing training*. This requirement ensures that the system actually provides the intended benefits of VR simulation over other existing training (simulated or otherwise). This is twofold; firstly, the simulation needs to use scenarios from real case studies (i.e., what you wish to train for) (Vaughan et al. 2016), and secondly, the simulation should provide a controlled environment that is low risk and safe for both the trainee and others present (Bielsa 2021, Bernardo 2017). Consideration should also be given to unintended consequences of low-risk training (e.g., trainee’s translation of the training experience may result in perception of real life risks becoming artificially low, particularly if the simulation was a no-fail scenario).

#### Usability (4.0)

*The system shall enable the user to achieve their goals with effectiveness, efficiency and satisfaction*. This requirement follows the ISO 9241-11 (International Organization for Standardization 2018) definition which covers both the effectiveness of the human–system interactions, but also various measures of usability. The extent to which usability was measured varied from study to study. Mäkinen et al. (2020b) reviewed different types of VR systems against ten user-experience (UX) dimensions (cited from Tcha-Tokey et al. (2018)) including usability and found that usability was the most commonly observed dimension. Furthermore, the studies they reviewed showed that usability underpins the other dimensions and has a great impact on learning effectiveness and overall learning experience (particularly for VR-HMDs). Chang et al. (2019b) also focused their study on the user experiences in gynecological VR training, measuring usefulness, perceived ease of use, enjoyment, controls and engagement. Similarly, Pedram et al. (2020) discussed usability as a combination of plausibility, ease of use and usefulness.

##### Plausibility

*The system shall provide credible scenarios*. Plausibility of the system involves the displays and procedures not only have fidelity but also be perceived as a credible scenario. The use of real-life operating room scenarios (or mock operating room settings), for example, should be used to convey plausibility (Lohre et al. 2020b). Clinical variation is also important, scenarios and patient populations will not be homogenous, and therefore, representations/simulations should also include such variation (Bernardo 2017).

##### Ease of Use

*The system shall enable the user to accomplish their goals*. (Huber et al. 2018a) (Pelargos et al. 2017b). Ease of use was discussed in numerous papers; however, there was a varied set of measures. Bernardo (2017)’s study found that box-trainers were more cost-effective, but VR training was more efficient.

##### Usefulness

*The users shall perceive the systems as being useful*. Perceived usefulness can be achieved when learners perceives that the training system can improve their task performance (Makransky and Petersen 2019).

### Learning mechanisms

Virtual reality (VR) offers a unique learning mechanism that enhances the learning experience by providing an immersive and interactive environment. VR technology enables learners to experience simulations that are otherwise impossible to replicate in real life, allowing them to acquire practical knowledge and skills. The immersive nature of VR also enhances learner engagement and motivation, leading to improved retention and transfer of knowledge. Additionally, VR can facilitate the development of spatial reasoning, problem-solving, and decision-making skills, which are crucial in many fields, including medicine, engineering, and aviation. In the next section, the requirements for learning experiences are covered and discussed.

#### Learning experience (5.0)

##### Immersion/sense of presence

*The system shall provide feeling of being immersed.* There is no clear definition for immersion (Skarbez 2017). Fox et al. (2009) define immersion as: “The psychological experience of losing oneself in the digital environment and shutting out cues from the physical world is known as immersion,” while Slater (1999) regards immersion as an objective characteristic of a VR and depends on the choice of hardware. Witmer and Singer (1998) defined presence as “the subjective experience of being in one place or environment, even when one is physically situated in another” (p. 226). Following Slater (1999) definition for immersion, to create a presence hardware needs to support and provide optimal refresh rates, frame rates, display sizes and display resolutions. Bracq et al. (2019) measured level of presence in the VR-based surgical training and reported expert users perceived higher presence than non-expert users. Mäkinen et al. (2020b) compared simulators with haptic device versus the simulator and find out that participants experience higher presence and a better learning experience with simulator plus haptic support.

##### Stress inducing and flow/enjoyment

*The system shall not cause more stress and be enjoyable.* One of the factors in assessing HMDs in medical education is perceived stress versus flow and enjoyment. Previous research shows that the experiencing stress and sickness during VR-based training can impede learning and training (Jensen and Konradsen 2018). It is unclear if these symptoms are related to beginners’ trying to familiarize themselves with the technology or if these symptoms continues may potentially adversely impact learning or education (Pedram et al. 2021)). As participants level of stress increases, it is more likely that they will develop a negative attitude toward the technology. VR learning environment should be design in a way to enhance the pleasure of the experience. Based on flow theory, when the task is realistic, and at an appropriate level of difficulty, the user becomes fully concentrated and in control of their activity that is when they lose track of time and become deeply involved with the training and training content and will ultimately experience less stress (Csíkszentmihalyi 1990).

##### Capability matching

*The system shall match device capability, surgical task and user proficiency,* although new technologies such as VR create an opportunity to enhance the quality of training and ultimately increase the accuracy and precision of surgical tasks (Rahman et al. 2020). The training platform must match the capabilities of a technology to the demands of a task. The technology must facilitate the training in a way to enhance users’ learning behavior, which is the determination of learning outcomes.

##### Social presence

*The system shall provide the feeling of social presence.* This requirement was not covered in the papers we reviewed; however, it is a crucial requirement when conducting collective training. Social presence refers to the user perception of the capability of the tool to foster the social aspect of the experience (Pedram et al. 2020). A system which supports Social Presence allows participants to interact with others in virtual space (Huber et al. 2018a).

##### Motion/simulator sickness

*The system shall not cause motion sickness.* This requirement ensures that user has a pleasant training experience. Trainees who experience simulator sickness will be distracted from the training and will not be able to concentrate on content, possibly resulting in negative learning experience, lower sense of engagement and presence and even lead them to withdraw from training (Pedram et al. 2021). Simulator sickness occurs due to the disconnection between the virtual and real experiences. The symptoms include nausea, vomiting and eyestrain (Kenngott et al. 2021b). One way of reducing discomfort is by introducing rest frames (Duh et al. 2004). A rest frame is any stationary object which helps VR technology users to distinguish which object is moving and which object is stationary. The Simulator Sickness Questionnaire (SSQ) developed by Kennedy et al. (1993) can be used to measure the individual level of simulator sickness.

#### Learner’s state of mind (6.0)

##### Self-efficacy

*The system shall enhance users self-efficacy.* Self-efficacy needs to be measured prior- and after-training session. Self-efficacy is defined as the trainee’s perceived capability to learn or perform an action. It is reported that there is a relationship between self-efficacy and learning experience; as trainees’ believe, they are able to complete and achieve the expected outcome; they have higher motivation to try harder during training (Makransky and Petersen 2019). As trainees get an opportunity to be exposed to number of scenarios and emergency situations, that can contribute in enhancing trainees’ self-efficacy (Barteit et al. 2021b).

##### Competition

*The system shall provide scoring or a competitive feature.* By providing incentive, score or feedback of trainees performance, it will encourage them throughout the training session to get involved and commit to the training task (Bracq et al. 2021). For example, Mehrotra and Markus (2021b) report on serious games and provide example of Kahoot® which the game had a time limits and provided score based on students’ performance. The result of the analysis indicated that by creating competitive environment, it resulted in learning gains and high participant satisfaction.

##### Learning from Mistakes

*The system shall enable users to learn from mistakes.* If VR training is to be a mechanism for reducing errors in real-life practice, then trainees need to understand the effects of the errors to learn from their mistakes.

##### Motivation (enjoyment, satisfaction, engagement, attention, challenge)

*The system shall provide motivation through enjoyment, satisfaction, engagement, attention and challenge.* Motivation is individual’s state of mind that drives their behavior. The extent learners feel presence and engagement, the more motivated they will be to perform the task (Makransky and Petersen 2019). Based on goal theory (Blumenfeld 1992), the training session to be successful and engaging the content of the training must be meaningful and include variety, diversity, challenge and control to the extend which does not cause cognitive load. One side effect of cognitive load is inattentional blindless, where trainees fail to see an object because it has not caught their attention (McKnight et al. 2020a; Chang et al. 2019b). Generally, when a given task displays variety and diversity and appropriate level of challenge (depending on participants’ level of expertise), trainees tend to engage better with the training. However, the reaction of the trainees to the challenge depends on their perception of the training material or environment. The quality of their engagement will increase if they perceive that what they are learning is meaningful. Meaningfulness has been defined as training and material that “makes cognitive sense” or/and creates “interest and value.” As part of learning process, learners desire for autonomy and being in control, competence and relatedness and are more likely to engage with the training to learn when they are in a learning situation in which they perceive that these needs are met.

#### Learning (7.0)

##### Learning outcomes

*VR shall enable defined learning outcomes.* According to Webster and Hackley (1997), ultimately it is the instructional introduction and implementation of technology, and not technology itself, that determines learning experience and outcomes. The technology must facilitate the training in a way to enhance users’ learning behavior, which is the determination of learning outcomes. (Pedram et al. 2020) As it has been reported by Bernardo (2017), ideal learning can occur under the following conditions: “feedback during training, repetitive practice, curriculum integration, range of difficulty level, multiple learning strategies, capturing of clinical variation, a controlled environment, individualized learning, defined outcomes, and validity” (p. 1026).

##### Assessment

*The system shall use measures for assessment.* Pithers (1998b) defines competencies as attributes which underlie successful performance. Learners’ competency can be assessed subjectively by having candidates rate their experience and performance, (Pedram et al. 2021) or objectively, where the system measures procedure duration, task completion rate or error rate. Assessments include technical surgical skills, while it also aims at identifying participants’ strengths, weaknesses and any areas for improvement for all skill levels. This can be useful in tracking development and acquisition of skills in surgical trainees, as well as assisting to maintain skill level in advanced surgeons (Pelargos et al. 2017b).

##### Subjective assessment

*The system shall collect subjective assessments.* In this technique, participants are asked to share their opinions about the conducted training, the approach of the training and the features impacting the process. These techniques provide insight on factors such as perceived joy, stress, satisfaction and other non-measurable factor and assist on reflect on training process where it is difficult to measure performance and training outcomes. This technique does not reflect on the extent of knowledge creation and of training to physical world (Nutakor 2008).

##### Objective assessment (procedural duration, task completion rate, error rate)

*The system shall use objective measures of learning.* With the improvement in the surgical simulators, objective metrics such as performance or knowledge measures can be incorporated into surgical simulators; this way competency can be assessed by standardized assessment tool (Breitkreuz et al. 2021a). When VR is used as part of an assessment, it can either measure procedural completion time, number of completed tasks, accuracy of using surgical devices, collisions between a learners' tool and simulated anatomy and efficiency of movement (Mao et al. 2021b, Bernardo 2017, Breitkreuz et al. 2021a) or measure technical and content-related knowledge through the application of structured assessment or curriculum-based assessment such as Knowledge Space Theory (KST) (Vaughan et al. 2016). However, many important aspects of effective surgical technique such as proper drilling and suctioning technique, maintenance of proper visibility of the surgical field and identification of anatomic structures have not yet been explored and incorporated into the simulator as performance metrics (Bernardo 2017).

##### Skills (psychomotor skills, social skills, task-specific skills, diagnostic skills, skills tracking)

Surgeons and procedural doctors require to acquire competence not only in technical skills (physical examination, manipulation of tools, psychomotor skills) but also the soft skills (communication, teamwork, leadership), cognitive skills (decision-making, situational awareness) and self-management skills (managing stress and coping fatigue) (Mehrotra & Markus 2021a, b). VR will allow hospitals to track surgeons or practitioners skills by evaluating their performance in a safe VR setting (Bernardo 2017).

##### Differential learning and curriculum design

As it has been reported by Bielsa (2021), mixed-modal training is proved essential and superior to the traditional Halsted approach regarding the acquisition of surgical skills. Simulation-based training can help trainees develop the required skills and knowledge in a more efficient way. The virtual environments enable learners to visualize relations and gain a first hand experience in virtual environment, which could never be possible otherwise, allowing to practice in a safe and technology-enhanced learning platform (Mehrotra and Markus 2021b). That said, the simulation should become a component of a curriculum in which the learners can use and benefit from a sequential and constructive learning process (Bernardo 2017). VR by offering an individualized learning opportunity enables trainees to receive training on a specific topic of interest or need. VR will enable trainees to have a repetitive practice on a range of scenarios of different difficulty level by incorporating clinical variation in the scenario in a controlled environment(Bernardo 2017). The aim is for practitioners to be exposed to broad range of possibilities and be able to transfer their virtual skills into the real life.

#### Trainer and feedback (8.0)

##### Trainer and feedback (corrective, explanatory and performance feedback)

As VR creates opportunity of repetitive training, trainees error and mistake can be identified and refined by feedback from the trainer or system (Bielsa 2021). It was highlighted by Fairen et al. (2020) that students and trainers reported on the importance of getting information from the application so trainer can provide feedback. These data could be on their motivation, level of interest and possible problems and mistakes they made (Fairen et al. 2020). However, there was disagreement between lecturers and students on when to provide the feedback. Students generally preferred to receive the feedback during the training and individually while the lecturers preferred to provide performance feedback right after the training sessions. It is reported that consistent feedback during the training can reduce the cognitive load for the learner and allow them to focus on the task (Breitkreuz et al. 2021a), while performance feedback is based upon direct observation or collected data by the simulator which allows trainers after the training to take trainees through the specifics of the tasks or its sub tasks. While there is no clear indication of when is the right time to provide feedback, Moreno et al. (2002) state that the form of the feedback is more important. The categorize feedbacks as: “corrective feedback,” where the learners are informed on if the performed task was right or wrong, and “explanatory feedback,” where the explanation is provided to learners on why the performed task was right/wrong. The result of their analysis reveals that trainees who received explanatory feedback outperformed the group who received corrective feedback in solving complex problems.

### Implementation considerations

#### Expertise (9.0)

The literature suggests that for successful implementation of a VR-HMD training system for medical education, 3 main types of expertise are needed:

##### VR developers

*VR experts shall be involved in the development of the system*. VR development needs a blend of skill-set and expertise from two fields: (1) computing and coding/software development and (2) human-centered design (human-centered interaction) (Papagiannakis et al. 2020). Gelardi (2020) (via Breitkreuz et al. (2021a)) discussed the time- and labor-intensiveness of the VR development as well as the additional graphics skills required to create the visuals. One of their key lessons learnt was the lack of experienced VR team impeding the speed and quality of the development—they noted limited funding and lack of access to qualified programmers to either produce the code or mentor their team to accelerate their technical VR knowledge.

##### Teaching/subject matter experts

*The system shall be developed with medical trainers or lecturers*. The medical trainers or lecturers should be involved in the development of the system providing the knowledge around the medical scenarios, anatomy/physiological landscape, technical skills and techniques in addition to driving the learning outcome requirements and integration into the curriculum. VR simulation should also include access to expert tutors, real-life clinical experience and learner-centered orientation (Bielsa 2021). (Fairen et al. 2020) both subject matter (anatomy) and teacher expertise required of the trainer.

##### Student technological expertise

*The system shall cater for users with a range of gaming experience and/or digital literacy*. Gaming experiences and perception of computing technology in general affect the trainee’s experiences in VR-mediated learning ((Pedram et al. 2022, Pedram et al.^2020^)). Fairen et al. (2020) describe *VR4Health*, a self-guided VR teaching platform that enables personalized learning for different levels of student expertise. Student expertise also presents a challenge in terms of measuring performance; students require digital literacy and some basic training to use the VR equipment and programmed controls in order to have sufficient skills to attempt any procedures within the VR environment.

#### Technology adoption (10.0)

This group of requirements is focused on the considerations that need to be taken into account for decision-makers to determine whether or not to adopt VR technologies into the wider medical educational training systems.

##### Affordability

*The system shall be cost-effective for the increased value expected*. With limited budgets, the affordability of new VR training systems is a major factor in the decision over whether to adopt the new technology. Breitkreuz et al. (2021a) reported that gaining funding for implementing VR into training systems was a challenge. Such systems must therefore achieve economies of scale (Pelargos et al. 2017b) while not exhausting the financial resources that have been dedicated to enhancing the curriculum (Mao et al. 2021b). A number of studies focused on technology concept demonstrators that utilized low-cost components such as a students’ existing smartphone as the VR display (Lopes et al. 2017a; Silva et al. 2018b; Xu et al. 2020; Masuoka et al. 2019b); however, these generally did not deliver value as the display quality and processing speed was too low (Xu et al. 2020; Masuoka et al. 2019b). Where others looked at reducing the cost of individual components, Huber et al. (2018a) spoke of the need to reduce the number of components to improve cost-effectiveness. (Lohre et al. 2020b).

##### Attitude to VR

*The system shall not increase negative attitudes to VR*. (Chang et al. 2019b) Scepticism (Breitkreuz et al. 2021a).

##### Training transfer

The use and interest in VR-HMDs for training have been increasing throughout this century (Jensen and Konradsen 2018), but a common complaint is the transfer of the technology into tangible training outcomes (Lohre et al. 2020b). The system must be both fit for purpose, but provide improvement over or alongside existing training mechanisms if it is to be a suitable candidate for adoption. Although certain skills acquisitions were advantageous in immersive VR, some applications had no advantage over less immersive training solutions (Jensen and Konradsen 2018; McKnight et al. 2020a). There are three main VR training evaluation methods: (1) performance measures, (2) self-reported measures, and (3) observations (Renganayagalu et al. 2021). A common limitation among the studies reviewed was the lack of measured outcomes, not just in the immediate, but over longer-term clinical performance (McKnight et al. 2020a), learning curves, skills retention and career development (Vaughan et al. 2016). At a meso-level, Mao et al. (2021b) address the lack of proven outcomes, aside of the learning outcomes, but from the wider healthcare system (e.g., patient safety and healthcare outcomes).

##### Easily available

*The system shall be easy to obtain for use*. Lopes et al. (2017a) discuss the need for low cost and easily attainable technology. As discussed in the *Affordability* requirement, many recent studies looked at the feasibility of commercial smartphones as the display and processor for a VR system. Utilizing a student’s existing technology assets (high availability) as an ad hoc VR resource has become an area of interest during COVID as medical teaching in tertiary institutions was constrained to remote methods of learning. In addition to ease of obtaining the technology, there is also a need to consider the distribution and scheduling of resources to ensure fair access to the system (for both learners and teachers).

##### Adherence to regulations

Any new system being adopted for use in the medical education field must adhere to the relevant regulations, e.g., medical image privacy, patient data, (Masuoka et al. 2019b), ethical implications for both users and patients (McKnight et al. 2020a) and HIPAA compliance (McKnight et al. 2020a). For VR-HMD being used in live procedures, the relevant regulatory requirements for medical devices for the healthcare organization and country of use must be met, including standards such as the safety and quality management of medical devices outlined in ISO 13485 (International Organization for Standardization 2016).

##### VR Support/expertise

*The system shall provide technical support for the VR components*. Support will be required for both the hardware and software components of the VR system. Breitkreuz et al. (2021a) discuss the need for an experienced VR team to develop, make on-going improvements, maintain and support the system.

#### Technology (11.0)

This group of requirements covers the minimum technological hardware and software needs (where stated from the literature review) or from the body of knowledge on human factors/limitations.

##### Display quality

*The display shall be of sufficient quality*. Huber et al. (2018a) discuss the aspects of immersion and sensation of presence in VR, stating that in terms of hardware needs, the frame rate, refresh rate, resolution and size of display should be optimized. Kenngott et al. (2021b) also describe the need for higher display resolutions, but for the purposes of reduced simulator sickness. Studies have shown that when resolution is too low (720p), it is difficult for users to distinguish small objects displayed on the screen accurately, and that at least 1080p is required to ease observation tasks where VR is provided using smartphone style devices (Lopes et al. 2017a). The pixel density of eye limiting resolution for normal vision is ~ 60p/°. Most mainstream Commercial Off-The-Shelf (COTS) VR-HMDs with integrated displays provide 1080p + resolution (or pixel densities between 10 and 25p/°).

##### Battery life

*For devices that are not powered through a tethered connection, the device shall have a battery life commensurate with the duration of the training session*. As technology has improved, there are more light-weight, non-tethered VR-HMD solutions coming onto the market (e.g., glasses-style VR/AR); however, battery life for these technologies is poor (McKnight et al. 2020a). The computational power required to run high-quality synthetic environments increases the battery usage, resulting in shorter battery life between charges.

##### Wearability

*The device shall not cause physical strain or long-term harm through prolonged wearing of the device*. Huber et al. (2018a) discuss a positive attribute of “low weight” for the technology stating that the ~ 500 g weight of HMDs has the potential to become uncomfortable. Studies showed that improper weight (Yan et al. 2018) and posture/balance while moving (Nilsson et al. 2015) can cause physical workload on the neck and increase musculoskeletal disorder risk. From related research on head-mounted loads, the maximum acceptable mass of the headset shall not exceed 1000 g (LeClair et al. 2018); however, the level of discomfort is correlated with increasing mass (Yan et al. 2018).

##### Freedom of movement

*The device shall not encumber the normal range of physical movement required for a task*. Huber et al. (2018a) discuss the benefits of wireless HMD technologies; Pelargos et al. (2017b) go further suggesting that lack of mobility of the VR user (alongside vision) as a major hindrance in the adoption of VR into the medical field at large. Tethered devices can restrict the physical movement of the user, either through physical wire obstruction or constraining distance moved through length of tether, both of which could cause suspension of perceived immersion, hazard to the user and risk of hardware damage.

##### Installation

*The hardware shall be easy to set up for a non-VR expert*. Intuitive setup of all the hardware (HMD, controllers, paired computers and other peripherals) is needed particularly if there is no dedicated VR facility, and the system is expected to be deployed in different locations with staffing of varying expertise. Huber et al. (2018a) describe the potential reduction in complexity through combining multiple computer set ups.

##### Portability

*The hardware shall be able to be moved and/or installed in different locations*. (Pelargos et al. 2017b).

##### Processing speed

*The system shall not be “glitchy” or have latency*. Slow processing speeds can cause technical glitches or latency (lag between the real-life controls/movements and the corresponding VR environment) (McKnight et al. 2020a). Kenngott et al. (2021b) also discussed the limitations of post-processing speed in the real-time creation of simulated models based on real anatomical data.

##### Security

*The system shall provide secure network access*. With the increasing ubiquity of wifi and network-based VR-HMDs, there is a need to ensure that the network access is secure (McKnight et al. 2020a); furthermore, all networked components of the training system (e.g., VR headset, link computers, cloud-based data servers, etc.) should adhere to information and data privacy and/or protection regulations of the organizations and their IT units.

## Requirements hierarchy and tailoring

Section 6 describes the set of eleven key top-level (Level 1) requirements for a generic VR-HMD training system for medical education; from these eleven top-level requirements, fifty-one sub-requirements (Level 2) were identified (described under bold headings in the previous section), and from these another thirty-nine requirements (Level 3) were identified. In this paper, we propose a systematic requirements-driven validation; the nature of these hierarchical decompositions of the requirements means that the validation of the system may be performed from the ‘bottom-up’ (i.e., satisfying the lower-level child or sub-requirements, builds the case for meeting the requirements of the parent), eventually resulting in a traceable landscape from which to claim full validity of the system against all its requirements (and thus proving it fit for purpose).

**Fig. 6:**
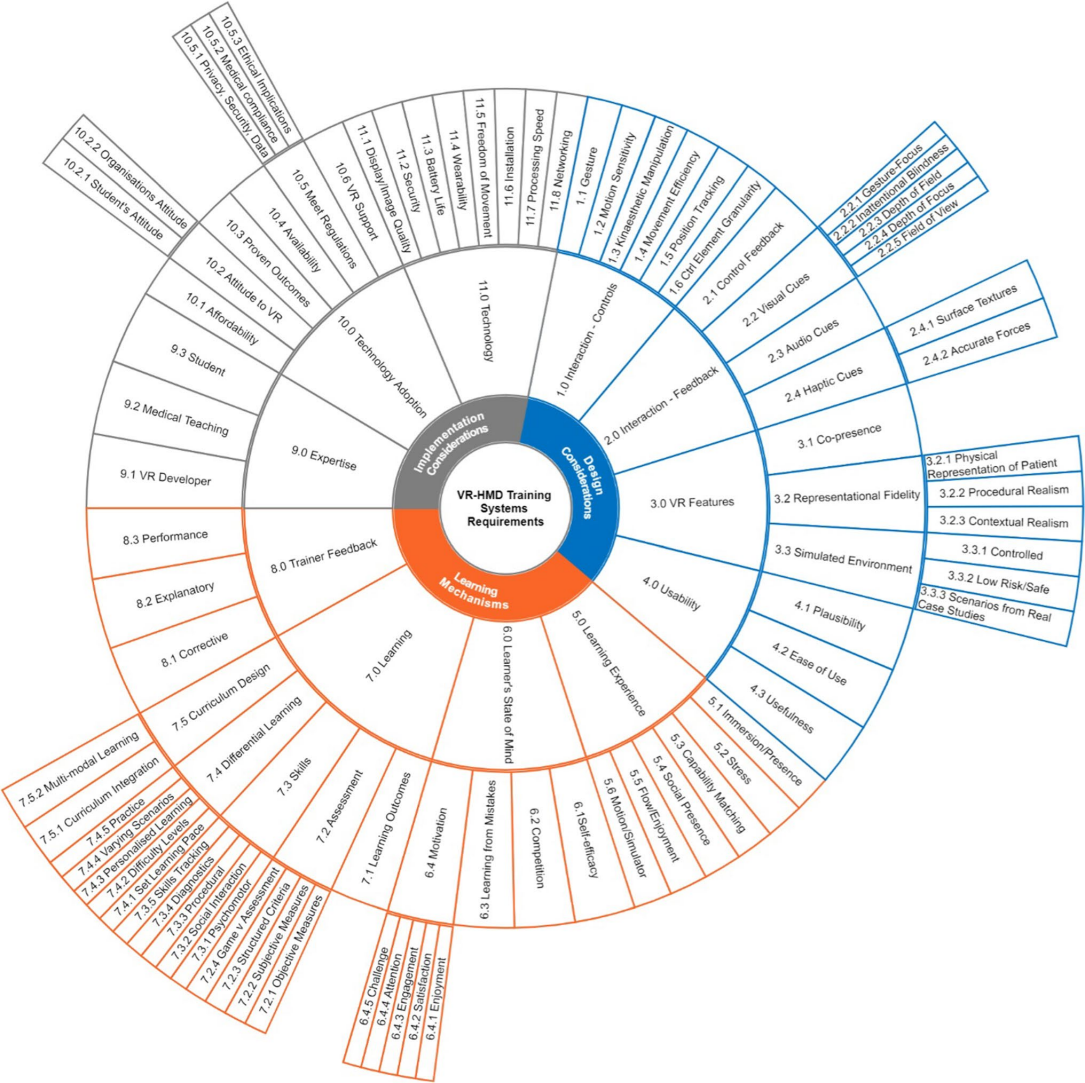
Requirements hierarchy wheel

These requirements have been drawn together for a generic VR-HMD training system within a wider medical education system (as described in Fig. 6); however, it is appreciated that not all contexts and applications will be the identical. For example, some requirements may be of lesser priority for a specific application or some may not be applicable. As explained in earlier sections, although the requirements appear dendritic in this hierarchy, there are linkages and interactions between them; these should be explored and decisions made around whether the interactions are of a positive or negative nature.

In order to effectively tailor these requirements, it will be essential for developers/adopters of VR educational systems to first identify the stakeholders, then develop the operational concept(s) of use (what the overall goal and context for the system will be). It is proposed that the framework and hierarchy of requirements outlined in this paper could be used as a reference or starting point to then confirm and stimulate needs and requirement discussions. Although each requirement has a suggested requirements starter sentence based on the literature, it is imperative that clear and concise requirements specifications are created, and these specifications should be tailored to capture the stakeholders’ needs or goals, be measurable, bounded by constraints, define the performance of the system and be verifiable (ISO/IEC/IEEE 29148 2018).

## Limitations and further work

This study is a starting point for developing a systematic evaluation approach to VR-based training programs. Despite all the constructive and comprehensive results generated by our research which can be generalized to various surgical training applications, some limitations have also been identified and further study will be needed to address these limitations:

- Our initial search was restricted to English-language articles, thus limiting the scope in studies reviewed.
- We have initially listed 31 papers to be included in this paper, but we were unable to locate three papers in the database and had to exclude them from the review process.
- This paper has focused on papers published in the past five years due to the greater availability of commercial VR solutions; future studies can include previous years to confirm the findings of this study.
- This study has systematically reviewed the application of HMD-VR for surgical training, and the design requirements reported here are mainly for the medical domain; further work is required to expand this research and report requirement for other domains of applications.
- The sample size for research studies included in this review is relatively small; half of the paper included in this study were review papers; this will limit the strength of conclusions that could be drawn.
- Furthermore, the keywords “need,” “must,” “shall” and “should” as indicators of need might not have been used to outline the requirement and future studies can expand the keyword search to capture any requirements which have not been included.

## Conclusions

This paper described a systematic literature review to draw conclusions on the contemporary state of VR-HMDs training systems for medical education. The findings indicated that there is a continued growth in VR training applications within the medical domain across a wide gamut of contexts and proposed learning outcomes, and that the acceleration of disruptive technologies such as VR shows increasing potential for training. The major challenges for the field of research are the ad hoc nature of the ever-growing collection of VR technology feasibility studies and reviews, and the lack of longer-term studies to investigate true learning outcomes and the need for validation of the holistic system (i.e., can education establishments be satisfied that the proposed adoption of the VR technology will enhance learning, be fit for purpose and yield value?).

The research outcome outlined in this paper proposes a requirements-driven approach (supported by the generic requirements framework hierarchy) to support validation efforts for new VR training systems such that they will successfully provide the functionality and value needed by their stakeholders. Therefore, the envisaged beneficiaries of this research are those stakeholders of the VR training system (the learner, teacher and VR providers); however, there is a wider set of stakeholders at the meso-level training system (their medical training organizations such as universities, colleges and teaching hospitals). Even with the best technological solution, care is required in adopting and implementing technological solutions into existing organizations so these meso-level stakeholders’ needs must also be considered. Ultimately, the end beneficiaries will be the healthcare systems and the patients under the care of the learners who will become the future workforce.

Although the requirements framework hierarchy is focused on training for medical education, similar approaches could be adopted for other VR training applications in other domains. Many of the requirements are transferable across any domain. It is envisaged that similar generic frameworks could be developed as references for different contexts.

The requirements-driven approach has not yet been fully applied as an exercise to validate a VR training system; however, the authors of this paper are currently undertaking a study into how the requirements hierarchy framework may be tailored to support the validation (and iterative design improvement decisions) for an existing VR tool for teaching clinical skills to medical students.

## Appendix

**Table:** 

Reference	Aim	Study design	Field	Pre-op planning or training	Subject of investigation/ Sample	Research method	Main findings
Barteit et al. (2021a)	To examine the effectiveness of AR, MR, and VR HMDs for medical education	Review paper	Medical education	Training	N/A	Seven medical databases (PubMed, Cochrane Library, Web of Science, Science Direct, PsycINFO, Education Resources Information Centre, and Google Scholar) were searched for peer-reviewed publications from January 1, 2014, to May 31, 2019	HMDs were most often used for training in the fields of surgery and anatomy where training with AR- and VR-based HMDs was perceived as salient, motivating, and engaging
Bernardo (2017)	This study explored the current and future roles and application of VR and simulation in neurosurgical training	Review paper	Neurosurgical Training	Training	N/A		VR and other simulations can reduce operative time and error by increasing confidence and wasted movements of the surgeon
Bielsa (2021)	To review the progress of VR simulation in plastic surgery (PS) training	Review paper	Plastic Surgery	Training	N/A	A systematic search of the literature was performed on PUBMED/MEDLINE with the following key words: (Simulation OR Virtual Reality) AND (Education OR Training) AND Plastic Surgery from January 1998 to September 2019	VR simulators are a promising tool for microsurgical training. While other surgical specialties are using simulation for both training and re-certification, reconstructive microsurgery has lagged. They conclude that experience with simulation in surgeries such as plastic surgery seems to be very low. Further efforts are required to include simulation in plastic surgery training curricula
Bracq et al. (2021)	To design a simulation scenario for “Error recognition in a virtual operating room” to improve situation awareness	Multiple groups–between-group design	Scrub Nurse SA training	Training	18 scrub nurse students and 8 expert scrub nurses	Participants reported any errors they observed. There were nineteen errors with various degrees of severity. Measures were retrieved from logs (number of errors, time for detection, movements) and from questionnaires (situation awareness, subjective workload, anxiety and user experience)	The training was more successful for those who felt more immersed in the virtual operating room than those feeling less immersed. Students detected more errors than experts highlighting the need for refresher trainings
Breitkreuz et al. (2021b)	To investigate the second-generation Virtual Reality Sterile Urinary Catheter Insertion Game (VR SUCIG)	Single group	Sterile Urinary Catheter Insertion	Training	46 nursing faculty and other qualified nursing and healthcare professionals participated	Participants were asked to play the VR game in private one-on-one game sessions. The game kept track of time, errors, and the number of times the sterile field was contaminated. The score and the time to task completion were visible on a virtual wall in front of the player. Further, System Usability Scale—Scores, User Reaction Survey (URS) was used	This paper summarize how the content, cultural variation and technical issues has negatively impacted users’ training experience
Chang et al. (2019a)	To design a new virtual reality (VR)-assisted training system, IFOREAL, for gynecology students	Multiple group—within-subject design	Gynecology	Training	100 undergraduate students participated	Participants were randomly assigned to professional (VR version) or consumer (Desktop) version The constructs included perceived usefulness (PU), perceived ease-ofuse (PEOU), perceived enjoyment (PE), perceived behavioral control (PBC), perceived internal control (PIC), attitude toward the system (ATT), satisfaction (SAT), confirmation (CON), engagement (ENG), attention (ATTEN), presence (PRE), behavioral intention to use the system (BIU for IFOREAL), and behavioral intention to use other VR training systems (BIU for VR training technology). Basic demographics, including age, gender, education level, domestic/intentional student, prior experience with VR technology, and level of personal innovativeness were also measured	The result of the study suggests that participants had a positive learning experience with both platforms. Desktop version provided more control while VR version created higher sense of presence. Female participants in general had a higher positive experience in comparison to the male participants
Chheang et al. (2021)	To improve virtual resection planning between surgeons in a remote or co-located environment. The system allows surgeons to define and adjust virtual resections on patient-specific organ 3D surfaces and 2D image slices	Single group		Pre-op Planning	3 surgeons	The guided exploration and interviews were conducted and system usability scale questionnaire (SUS) and presence questionnaire (IPQ) was used	The participants stated that the system might help to visualize and assess the spatial relationship between major vessels and the resection volume and, thereby, help in the identification of safety–critical areas prior to surgery. However, the participants emphasized that the tool would be primarily beneficial in cases that are challenging for a surgeon, i.e., either surgically complex cases or cases in which the surgeon has limited experience
Corrêa et al. (2019)	To review the state-of-the-art in virtual needle insertion training simulation based on haptic interaction	Review paper- Needle insertion training	Needle Insertion	Training	N/A	The scientific search databases used were IEEE Xplore (Institute of Electrical and Electronics Engineers), ACM Digital Library (Association of Computing Machinery), and Scopus	Reports on the need of haptic interfaces for needle insertion training in the healthcare area. This is crucial that a suitable set of learning tasks is designed, with proper task support
Fairén et al. (2020)	To design an application as a self-learning tool to allow students to directly inspect 3D models of several human organs using VR	Multiple group	Anatomy	Training	6 teachers of human anatomy and 18 students	The questionnaire, to measure: (1) Ease of use, (2) Self-learning usage and (3) Teacher helping usage	They conclude that VR4Health is a tool that is perceived by users as an easy-to-use tool for self-learning of human anatomy and physiology and that covers the need for learning support manifested so for students as well as for teachers
Hattab et al. (2021)	To evaluate the use of VR by comparing the recall of spatial information in two learning conditions: a head-mounted display (HMD) and a desktop screen (DT)	Multiple groups–between-subject design	Surgical planning	Pre-op Planning	56 student and medical staff	Authors examined whether participants would accurately recall the internal structure of two rendered models, a pyramid and a liver, experienced either via a HMD or a DT	The results revealed that the learning condition (HMD or DT) did not influence participants’ memory and confidence ratings of the models shown in VR. In contrast, the model type, whether the model was a liver or a pyramid, significantly impacted participants’ memory about the internal structure of the model
Huber et al. (2017b)	To observe user experiences and performance scores using a new combined highly immersive virtual reality (IVR) laparoscopy setup	Single group	laparoscopic Surgery	Training	10 members of the surgical department	Participants performed three tasks on a VR simulator. Questionnaires on immersion and motion sickness were used for the study	Participants’ times for fine dissection were significantly longer during the IVR session compared to traditional approach and also participants had higher error rate error in IVR while performing cholecystectomy task. None of the participants reported motion sickness and they experienced a high level of exhilaration, rarely thought about others in the room, and had a high perceived sense of presence in the IVR
Huber et al. (2018b)	To describe the process of developing a highly immersive VR simulation for laparoscopic surgery	Multiple group	Laparoscopic Surgery	Training	16 participants	The level of perceived immersion in VR, the attention to the environment and exhilaration of the participant was measured	The authors reported a proof of concept for the technical feasibility of the custom laparoscopic VR-HMD setup however, future technical research is needed to improve the visualization, immersion, and capability of interacting within the VR environment
Kenngott et al. (2021a)	To evaluate the potential of VR environment for liver surgery that integrates all relevant patient data from different sources for planning and training	Single group	Liver Surgery	Pre-op Planning and Training	158 participants (57 medical students, 35 resident surgeons, 13 attending surgeons and 53 nurses)	After using the system, a 10-item online questionnaire was filled out by the participants using the Likert scale	Participants generally agreed that complex cases in particular could be assessed better and faster with VR than with traditional 2D display methods. The highest potential was seen in student training, resident training, and clinical routine use. Least potential was seen for nursing training
Lohre et al. (2020a)	To characterize previous uses of VR in shoulder and elbow surgery in preoperative, intraoperative, and educational domains including trauma and elective surgery and to provide recommendations and framework research surrounding iVR in shoulder and elbow surgery	Review paper	Elbow Surgery	Training	N/A	N/A	Orthopedic surgery, and specifically shoulder and elbow surgery, have demonstrated promising early trials with virtual preoperative planning, and intraoperative adjuncts, particularly with fracture management. The largest focus of VR has been on surgical education and simulation, particularly that of arthroscopic trainers for shoulder surgery
Lopes et al. (2017b)	This paper has twofold research questions 1. Can a low cost simulator be created to enable medical students to learn at home 2. Can they train students to visually recognize different types of surgical instruments	Single group	Surgical Instrumentation Training	Training	19 users ( 8 were health students or professionals, 2 were heart surgeons, 1 was a resident doctor, 4 were medical students and 1 was a nursing student)	Used the Leap Motion input device/gesture recognition alongside a user's existing mobile phone display. Used Sauro and Lewis PSSUQ for usability assessment	Usability assessment showed most participants did not suffer from cybersickness but highlighted possible visual quality of the display
Mäkinen et al. (2020a)	To identify what kinds of VR technologies are used for learning in healthcare practice & education? and what kinds of UX were there when using the VR technologies?	Review paper	Healthcare education	Training	N/A	Integrative review (so empirical and theoretical) performed in 2019, based on 26 articles published between 2002 and 2019	Review found that of the three different VR technologies, haptic device simulators were the most commonly used in healthcare education, and HMDs were the least used. HMDs enabled observation of all ten components of UX for immersive virtual environments (presence, engagement, immersion, flow, usability, skill, emotion, experience consequence, judgment and technology adoption)
Mao et al. (2021a)	To examine the literature on the effectiveness of iVR for surgical skills acquisition	Review paper	Surgical Training	Training	N/A	Systematic lit review using PRISMA	Immersive VR-trained groups performed 18% to 43% faster on procedural time to completion compared to control group Training studies tend to be short in duration, so the effects of iVR training throughout a residency program are not reported
Masuoka et al. (2019a)	To investigate the issues that arise while observing a 3D model of an organ (taken from 3D slice models from scans)	Single group	Anatomy	Training	17 medical students	Google cardboard with Galaxy smartphone and valuated performance and usability	Favorable response to technology, but low score for motion sickness and eye fatigue Poor hardware/smartphone performance and visual quality
Matthews, S., Wood, K., Uribe-Quevedo, A., Jaimes, N., Dubrowski, A., Kapralos, B., Alam, F. and Rojas, D	Aim was to better understand how objects of interest are captured within HMD eye/gaze tracking	Single group	Cardiac training	Training	Five participants without any previous knowledge of cardiac auscultation procedure	Compares HMD ray casting v cone casting for eye tracking data. The first asked how well they felt they understood about different parts of the anatomy in the model. The second was the usability	Participants spend more time browsing a scene than specific instruments
McKnight et al. (2020b)	To investigate use of VR/AR for orthopedic surgery and the challenges for HMD usage	Review paper	Orthopedic surgery	Training	N/A	Reviewed studies using ODG R7 glasses, google glass, and Vuziz M300 glasses	While the hardware is advanced, there is still much work to be done in developing software that allows for seamless, reliable, useful integration into clinical practice and training
Mehrotra and Markus (2021a)	To explore the role of emerging simulation technologies globally in craniofacial training of students and residents in improving their surgical knowledge and skills	Review paper	Cranio-facial surgical training	Training	N/A		Simulation will be used alongside existing curriculum and cannot replace mentor–mentee relationships
Pelargos et al. (2017a)	To analyze current uses of AR/VR in neurosurgery and emerging applications. In particular, its use in pre-operative rehearsal of complex procedures	Review paper	Neurosurgery	Pre-op Planning	N/A	Used PubMed	VR and AR have the potential to transform neurosurgery by increasing the overall efficiency of training and treatment delivery, effectively These technologies can help mitigate patient risk and decrease the chances of surgical error by allowing neurosurgeons to learn and rehearse procedures in a zero-risk environment improving patient outcomes
Plotzky et al. (2021)	To identify the types of educational VR simulations have been developed for nurse education and the to identify design and implementation elements to provide effective learning scenarios	Review paper	Nurse education	Training	N/A	PRISMA	The necessity of need of including haptic devices, psychomotor skills and social interaction to teach soft skills
Rahman et al. (2020)	To report surgically relevant applications for which head-mounted display (HMD) use is reported	Review paper	Surgical application	Pre-op Planning	N/A	PRISMA	HMDs in education/training were 10% of surgical applications found, but more common to see them used intraoperatively (by the trainer sharing what they see with the trainee or vice versa). The quantitative results frequently involved time to complete surgical tasks, success rates, and relative precision
Silva et al. (2018a)	To investigate the application of virtual reality (VR) and related technology for clinical cardiac practice	Review paper	Cardiovascular applications	Training	N/A	Doesn’t discuss any method by which they searched for applications	Discusses 2 applications for educational cardiovascular training
Vaughan et al. (2016)	To investigate what existing orthopedic training simulators are available?	Review paper	Orthopedic surgery (particularly hip and knee)	Training	N/A	Mostly looked in PubMed Doesn't state any further filtering process	The conventional master-apprentice learning model for surgical training is inefficient Simulators are being increasingly validated for assessment as well as training There are some limitations inherent with commercial haptic devices
Xu et al. (2020)	To investigate whether the mobile VR version has a more immersive experience and portability compared to the AR Smartphone and to investigate whether the AR version produces a better mobile experience and is more suitable for cooperation and communication within the educational setting	Multiple groups–between-subject design	Anatomy for doctors and vets	Training	7 senior anatomy lecturers participated	Comparison between VR HMD and AR smartphone systems, in the trials participants were surveyed on general feeling, immersive level, motivation and feeling of control	They found the AR device did not have a performance as impressive as VR, but AR had high portability and communicability
Yamazaki et al. (2021)	To provide complex anatomy model via HMD for education for pre-op planning	Multiple groups–between-subject design	Anatomy	Pre-op planning	11 participants	Developed software to use CT scan slicing to make 3D bone models which then fed into HMD asked about the validity of the HMD model (mixture of specialists and trainees), participants completed questionnaire about usability and whether tool is fit for purpose	They reported that 3D HMD model was found to be superior to 2D models. HMDs enabled users to manipulate the model (flying inside the 3D model) adding to spatial learning as well as identification of anatomical features

## Declarations

### Data availability statement

The data that support the findings of this study are available upon request from the corresponding author Dr Shiva Pedram (spedram@uow.edu.au).
